# Prenatal genetic diagnosis associated with fetal ventricular septal defect: an assessment based on chromosomal microarray analysis and exome sequencing

**DOI:** 10.3389/fgene.2023.1260995

**Published:** 2023-11-24

**Authors:** You Wang, Ru Li, Fang Fu, Ruibin Huang, Dongzhi Li, Can Liao

**Affiliations:** ^1^ The First School of Clinical Medicine, Southern Medical University, Guangzhou, Guangdong, China; ^2^ Department of Prenatal Diagnostic Center, Guangzhou Women and Children’s Medical Center, Guangzhou Medical University, Guangzhou, Guangdong, China

**Keywords:** ventricular septal defect, chromosome microarray analysis, exome sequencing, prenatal diagnosis, fetus

## Abstract

**Objective:** In the study, we investigated the genetic etiology of the ventricular septal defect (VSD) and comprehensively evaluated the diagnosis rate of prenatal chromosomal microarray analysis (CMA) and exome sequencing (ES) for VSD to provide evidence for genetic counseling.

**Methods:** We carried out chromosomal microarray analysis (CMA) on 468 fetuses with VSD and exome sequencing (ES) on 51 fetuses.

**Results:** In our cohort, 68 (14.5%) VSD fetuses received a genetic diagnosis, including 61 (13.03%, 61/468) cases with chromosomal abnormalities and seven (13.7%, 7/51) cases with gene sequence variants. The detection rate of total pathogenic and likely pathogenic gene variations in the non-isolated VSD group (61/335, 18.2%, 55 by QF-PCR/karyotype/CMA + 6 by ES) was significantly higher than that in the isolated VSD group (7/133, 5.3%, 6 by QF-PCR/karyotype/CMA + 1 by ES, *p* = 0.000). The most common copy number variation (CNV) was 22q11.2 microdeletion syndrome. Additionally, we found six previously unreported variants, which expanded the variation spectrum of VSD-related genes.

**Conclusion:** In this study, CNVs and sequence variants were found in 13.03% and 13.7% of cases, respectively. ES can be recommended for fetuses with VSD without chromosome abnormalities and pathogenic CNVs, especially those that are combined with other ultrasound abnormalities.

## Introduction

Ventricular septal defect (VSD) is a frequent congenital heart disease (CHD), making up 26% of all CHDs (Ferencz et al., 1987). Its incidence rate among live births is 3.5/1000 (Hoffman and Kaplan, 2002). VSD can be due to environmental factors, genetic factors, or can be multifactorial. Genetic factors mainly include aneuploidy, chromosomal rearrangements, copy number variants, and sequence variations (Newman, 1985). Non-genetic causes can be identified in 2% of CHD cases, while 20%–30% of CHD cases can be traced back to genetic causes (Cowan and Ware, 2015). Qiao et al. reported that VSD is a type of CHD most often associated with a genetic cause, and 36.8% of VSDs are associated with genetic factors (Qiao et al., 2021). Although most VSDs are repairable and patients can achieve good long-term prognoses under optimized surgical and medical care conditions, the prognosis is unsatisfactory for some patients suffering from VSDs with related genetic abnormalities (van Nisselrooij et al., 2020; Mone et al., 2021). Therefore, the prenatal definition of genetic abnormalities is very important in the diagnosis of VSD, as it can provide more accurate and appropriate genetic counseling, which can affect the decisions of parents on the continuation/termination of pregnancy, prenatal monitoring, and perinatal care.

Fetal structural abnormalities are indicators for invasive prenatal genetic testing (Fu et al., 2022). Fetuses with structural abnormalities have a higher incidence of aneuploidy, chromosomal rearrangements, and sequence variations (Fu et al., 2018). Conventional karyotype analysis is an effective technique to identify chromosomal rearrangements, with a diagnostic rate ranging from 5.4% to 15.5% (Hanna et al., 1996; Beke et al., 2005). However, G-banding karyotype analysis has low resolution and is time-consuming and laborious. The detection rate of small genomic deletions and duplications increased by up to 10% following the development of array-based molecular cytogenetic techniques, such as CMA, which could not be detected via standard structural malformation fetal karyotype analysis (Hillman et al., 2013; Liao et al., 2014). CMA has high resolution and a short turn around time. In patients with post-natal and prenatal CHDs, it can identify aneuploidy, chromosomal rearrangements, and copy number variations (CNVs). Pathogenic CNVs are detected in 7%–36% of CHD patients (Fu et al., 2018; Wang et al., 2018). For the majority of fetuses with structural abnormalities, the underlying causes of abnormalities are unclear before genetic testing. As a significant advancement in next-generation sequencing (NGS), exome sequencing (ES) is an effective tool for assessing post-natal patients. This detection technology is used for prenatal diagnosis (Best et al., 2018). In addition to improving diagnostic rates, using ES for assessing a large sample size can analyze single nucleotide variations (SNVs)/insertions and deletions (indels) in the gene coding regions and help in the identification of novel pathogenic genes or novel variants in well-known genes in VSD patients (Sifrim et al., 2016; Jin et al., 2017; Fu et al., 2018; Lord et al., 2019; van Nisselrooij et al., 2020). Three extensive studies have shown that ES can provide an increased diagnostic rate of 8.5%–11.6% for fetuses with abnormal ultrasound findings, normal karyotype, and negative CMA results (Lord et al., 2019; Petrovski et al., 2019; Fu et al., 2022). A recent study on prenatal CHDs showed that the diagnostic rate of ES was 20% (6/30) (Westphal et al., 2019).

In the present research, we used CMA and ES to assess the detection efficiency of fetuses with VSD at the chromosomal (aneuploidy), sub-chromosomal (microdeletion/microduplication), and single gene (point variants) levels and evaluated perinatal prognosis to facilitate more accurate genetic counseling in clinical practice.

## Materials and methods

### Participant recruitment and sample collection

The Institutional Review Committee of the Ethics Committee of our organization gave its approval to this investigation. The study’s participation was approved by all parents, who provided their signed informed consent. We retrospectively studied 468 fetuses with VSD diagnosed by prenatal ultrasound in Guangzhou Women and Children’s Medical Center from September 2012 to September 2022. All fetal phenotypes were based on the results of prenatal ultrasound examinations. According to the standard fetal echocardiography guidelines from the American Society of Echocardiography (Sacks et al., 2018), all fetuses had complete 2D echocardiography with color flow and spectral Doppler imaging. Images were obtained utilizing a 1–5 MHz abdominal transducer on a Philips ultrasound machine (i.e.,33; Philips Medical Systems, Andover, MA).

The inclusion criteria of this study included fetuses diagnosed with VSD by prenatal ultrasound and with or without other structural abnormalities. The exclusion criteria included known infected fetuses, twin or multiple pregnancies, or exposure to known teratogenic drugs. We divided the cases into two large groups: non-isolated VSDs (fetuses with sonographic soft markers, other cardiac anomalies, or extracardiac structural anomalies) and isolated VSDs (fetuses with VSDs as the only cardiac defect). Soft markers are minor ultrasound findings identified in the mid-trimester of pregnancy that most commonly do not represent a structural abnormality and may be normal variants that most commonly do not represent a structural abnormality and may be normal variants but are noteworthy because of their association with an increased aneuploidy risk. These soft markers include choroid plexus cysts, absent/hypoplastic nasal bone, thickened nuchal folds (excluding the first trimester increased nuchal translucency), single umbilical artery, echogenic intracardiac focus, and echogenic bowel. In addition, congenital heart disease(CHD) was classified using the method described by Botto et al., 2007. We classified single ventricle, or multiple heart anomalies (involving three or more defects) as complex CHDs in our study.

Through the ultrasound system and the database of medical records, we gathered all clinical information about the patients, including maternal characteristics (maternal age, previous reproductive history), paternal and maternal medical history, the medical history of other family members (The parents had no clinical phenotypes except for one pregnant woman with VSD treated surgically, one pregnant woman and one husband with epilepsy, according to their routine physical examination reports.), gestational age when problematic cardiac abnormalities first appeared, ultrasound findings, the reason for referral to ultrasound examinations, nuchal translucency, results of a screening test for open neural tube defects, invasive testing indications, genetic testing results, outcomes of pregnancy, and *postpartum* treatment (if needed). Prenatal genetic testing is advised following comprehensive genetic counseling. The invasive procedures’ possible benefits and risks were conveyed to pregnant women and their families.

## Genetic testing tools

### Karyotype analysis

After obtaining a signed informed agreement, quantitative fluorescence polymerase chain reaction (QF-PCR) by utilizing a multiplex ligation-dependent probe amplification (MLPA) kit was used to analyze all 468 fetal samples to rule out the possibility of maternal cell contamination and swiftly identify aneuploidy including chromosomes 21, 18,13, X, and Y (Guangzhou, Darui Biotechnology Co., Ltd, Guangdong, China). Subsequently, standard laboratory procedures were followed to identify overall chromosomal abnormalities utilizing conventional G-banding karyotype analysis (550-band resolution).

### Chromosomal microarray analysis

We performed CMA in all 468 fetuses. For analyzing fetal samples with normal karyotypes, we followed the instructions of the manufacturer and used the Affymetrix CytoScan HD/750K array (Affymetrix, Santa Clara, CA, United States) to conduct whole-genome high-resolution microarray analysis to examine the submicroscopic genomic imbalance. The array included several single nucleotide polymorphism arrays (SNP arrays) and array-based comparative genomic hybridization (aCGH) platforms; the resolution was 10 kb and 100 kb, respectively. GRCh37/hg19 genomic locations were evaluated after construction. The specifics of this procedure are provided elsewhere (Cheng et al., 2022). Following the guidelines of the American College of Medical Genetics (ACMG), CNVs identified by CMA were classified as likely benign or benign, variants of unknown significance (VUS), likely pathogenic (LP), and pathogenic (P) (Riggs et al., 2020). Next, VUS, lpCNVs, and pCNVs were recorded, however, likely benign or benign variants were not considered. In total, 468 fetuses were assessed by CMA. A secondary technique, such as quantitative real-time PCR (qPCR) or fluorescent *in situ* hybridization (FISH), was used to confirm all *de novo* CNVs identified by CMA. If a clinically significant variation or VUS was identified in samples, parental CMA was recommended for these couples to facilitate the interpretation of CNVs and identify inheritance patterns.

### Exome sequencing

In addition to karyotyping and CMA, ES was offered as a research-based adjunct to prenatal diagnosis. Trio-WES tested the parents and fetuses with negative karyotype and CMA results. After the parents provided written informed consent, following the manufacturer’s instructions, the Agilent SureSelect human exome capture probes (V6, Life Technologies, Carlsbad, CA, United States) were used to enrich the DNA samples. To produce 150 bp paired-end reads, the DNA library was sequenced on the Illumina HiSeq2500, HiSeq Xten, or NovaSeq platforms. Supplementary File S1 contains comprehensive information on the analysis and interpretation of data on ES. Briefly, local reference and in-house pipeline samples were used to analyze the original fastq data (more than 10,000 people, involving healthy individuals and patients) and included information on mapping, realignment, variant invocation, quality control, variant filtering, annotation, gender, and family lineage confirmation. The variations were explained following the ACMG sequence variant guidelines (Richards et al., 2015). Supplementary File S2 shows the data analysis flowchart. Our positive findings included pathogenic and likely pathogenic variations. All diagnostic genetic variations were confirmed by Sanger sequencing.

### Clinical follow-ups

Pregnancy outcomes included live birth, neonatal death, and termination of pregnancy. We completed clinical follow-up assessments through electronic medical records and telephone records in the 6 months after birth and conducted routine follow-up evaluations every year. If any abnormality was found in the follow-up examination, we asked the pediatrician to further evaluate the case.

### Statistical analysis

The differences between groups were determined by performing Fisher’s exact tests or Chi-squared tests in SPSS 25.0 software (IBM, Armonk, NY, United States). All group differences were considered statistically significant at a *p-value* < 0.05.

## Results

### Characteristics of the cohort

**FIGURE 1:**
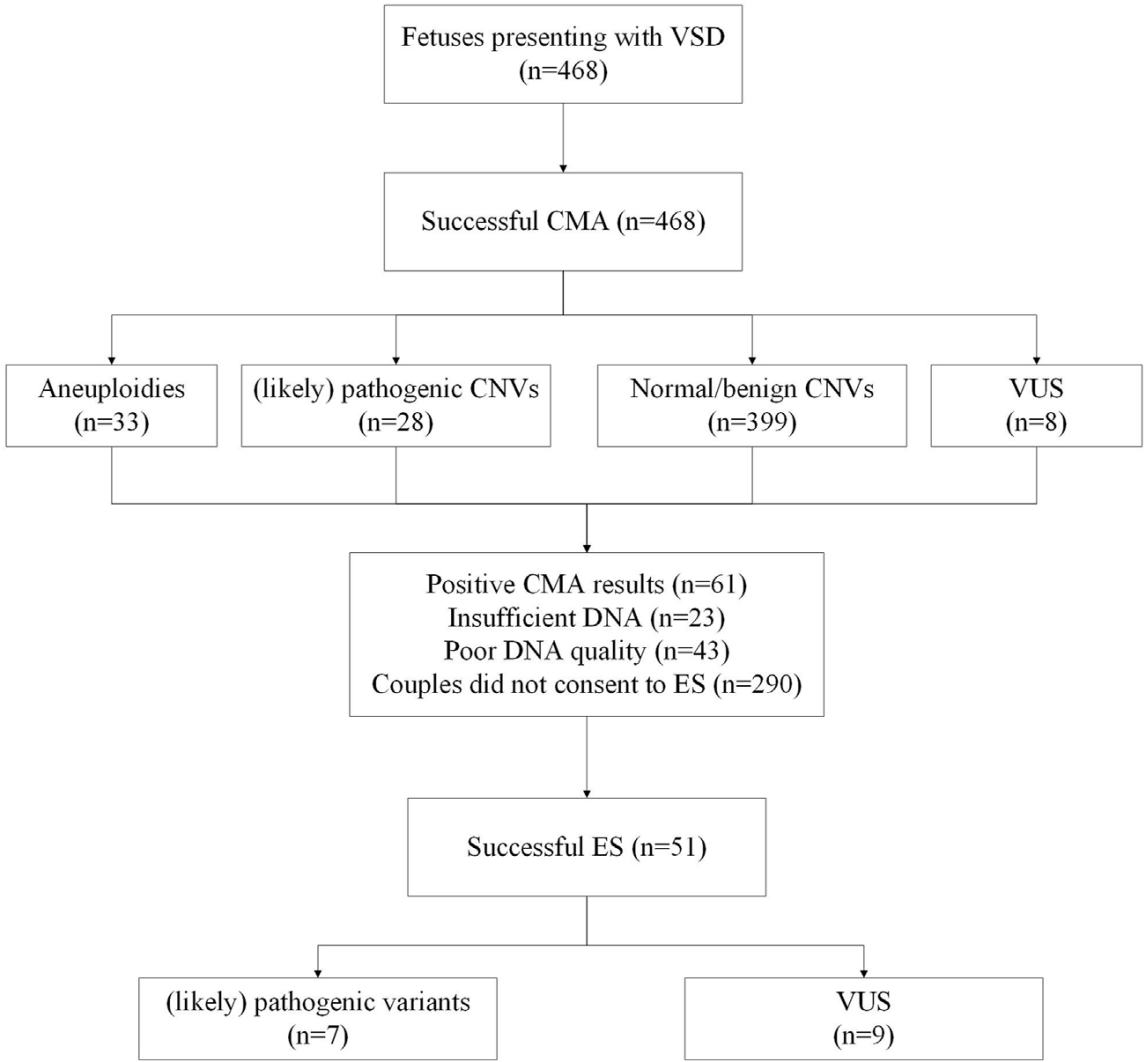
Flowchart of genetic analysis progression in a cohort of fetuses with VSD. CMA, chromosomal microarray analysis; VUS, variants of unknown significance; ES, exome sequencing.

In total, 468 pregnant women with suspected fetal VSD underwent invasive prenatal diagnosis in our center from September 2012 to September 2022. Figure 1 shows the genetic analysis procedure. The average maternal age (MA) was 32.7 years (range 20.2–47.3 years), and the median gestational age (GA) was 29.15 weeks (range 12.5–38.4 weeks). Most VSD cases were diagnosed in the second trimester (323/468, 69.0%) and underwent amniocentesis, while the remaining 145 cases (31.0%) were identified in the third trimester and underwent percutaneous umbilical cord blood sampling. Of these cases, 273 (58.3%) were male, and 195 (41.6%) were female. Muscular VSDs were found in 270 cases (57.7%), and perimembranous defects were found in 198 cases (42.3%). The median initial defect size was 2.1 mm (1.7–2.9 mm) in the muscular group and 2.6 mm (2.1–3.3 mm) in the perimembranous group (*p* = 0.000). These VSD cases were categorized into isolated VSD (*n* = 133) and non-isolated VSD (*n* = 335) based on whether they were combined with other structural abnormalities. We found that 198 cases had other intracardiac abnormalities, and 180 cases had extracardiac abnormalities, 43 cases with both intracardiac and extracardiac findings. The information on fetal abnormalities associated with VSD detected by ultrasound is presented in Table 1.

**TABLE 1:** Associated anomalies identified sonographically in VSD fetuses.

Associated anomalies	Number
Soft markers	26
Choroid plexus cysts	4
Absent/hypoplastic nasal bone	9
Thickened nuchal folds	7
Single umbilical artery	2
Persistent left superior vena cava	1
Echogenic intracardiac focus	2
Echogenic bowel	1
Cardiovascular system	197
Complex CHD	11
Single ventricle	4
Single ventricle + transposition of great arteries	5
VSD + Pulmonary atresia + Aortic coarctation	2
Simple CHD	186
VSD + atrial sepetal defect	17
Pulmonary stenosis	21
Ebstein anomaly	12
Pulmonary atresia + right aortic arch	33
Hypoplastic right heart	14
Double chamber right ventricle	11
Aortic coarctation	51
Aortic stenosis + right aortic arch	11
Aortic stenosis + aortic coarctation	6
Hypoplastic left heart	10
Renal anomalies	40
Enlarged kidneys	15
Pyelectasis	4
Duplex kidney	2
Renal cysts	11
Hydronephrosis	2
Multicystic dysplastic kidney	6
Skeletal system	81
Butterfly vertebra	14
Polydactyly	11
Hemivertebrae	15
Short femur	24
Clubfoot	17
Amniotic fluid anomalies	22
Polyhydramnios	15
Oligohydramnios	7
Gastrointestinal system	5
Hepatomegaly	2
Small stomach	1
Ascites	2
Central nervous system	17
Ventriculomegaly	4
Arachnoid cyst	3
Microcephaly	8
Macrocephalus	2
Others	16
Fetal growth restriction	14
Hydrops fetalis	2

CHD, congenital heart defect.

We carried out CMA on 468 fetuses with VSD and ES on 51 fetuses. CNVs and sequence variants were found in 13.03% and 13.7% of cases, respectively. Among them, 68 cases (14.5%) of fetuses with VSD underwent genetic diagnosis, which included 61 cases (13.0%, 61/468) with chromosome abnormalities detected by QF-PCR/karyotype/CMA and seven cases (13.7%, 7/51) of sequence variants detected by ES. Table 2 summarises the detection rate of (likely) pathogenic variants of fetuses with VSD.

**TABLE 2:** Diagnostic genetic variants that were identified in fetuses with VSD.

Case	Ultrasound findings	Gene (OMIM ID)	Reference sequence	Chromosomal locus (GRCh37/hg19)	Nucleotide/Protein change	Present in gnomAD/Inhouse DB	Inheritance and ACMG classification	Definite diagnosis	Outcome
1	Tricuspid atresia, single ventricle VSD	NOTCH2 (600275)	NM_024408.3	chr1:120539620-120572610	Exon 2–4 deletion	NO/NO	*De novo*, AD, P (PVS1+PS2+PM2) REVEL^#^: 0.847	Hajdu Cheney syndrome, Alagille syndrome type 2	TOP
2	Kidney agenesis, duplication of kidney, VSD	FANCI (611360)	NM_001113378.2	chr15:89848570	c.3187–2A>G	Yes (0.0001503 *)/NO	Het, Mat, AR, LP (PS2+PM1+PM2+PP3) REVEL: 0.779 Variation ID: 2445698	Fanconi anemia, complementation group I	Live birth
				chr15:89847103	c.3015G>C (p. Gln1005His)	NO/NO	Het, Pat, AR, VUS (PM1+PM2) REVEL: 0.783		
3	Isolated VSD	CHD7 (608892)	NM_017780.4	chr8:61763111	c.5464G>A (p. Gly1822Ser)	NO/NO	Het, *De novo*, AD, LP (PS2+PM1) REVEL: 0.847 Variation ID: 2041527	CHARGE syndrome; HH5	Live birth
4	Duplication of kidney, HEK, VSD	KMT2D (602113)	NM_003482.3	chr12:49440501	c.4308delC (p. Ser1437ProfsTer69)	NO/NO	Het, *De novo*, AD, P PVS1+PS2+PM2 REVEL:0.838	KABUK1	TOP
5	FGR, VSD	DOCK6 (131320)	NM_020812.3	chr19:11356555	c.807-1G>A	Yes (0.00008442*)/NO	Het, Pat, AR, LP (PVS1+PM2) REVEL: 0.847	AOS2	TOP
				chr19:11363153	c.377 + 5G>A	Yes (0.0002226*)/NO	Het, Mat, AR, VUS (PM1+PM2+PP3) REVEL:0.578		
6	PA, VSD	PTPN11 (176876)	NM_002834.5	chr12:112910827	c.836A>G (p. Tyr279Cys)	NO/NO	Het, *De novo*, AD, P (PVS1+PM2+PP4) REVEL: 0.858 Variation ID: 13328	NS1, LPRD1	TOP
7	Bart’s dropsy embryo, VSD	PTPN11 (176876)	NM_002834.5	chr12:112926887	c.1507G>A (p. Gly503Arg)	NO/NO	Het, *De novo*, AD, P (PS2+PS3+PM1+PP3) REVEL: 0.882 Variation ID: 40559	NS1, LPRD1	TOP

*: Allele frequencies of East Asian populations;^#^: Bioinformatics software predicted that the mutation would destroy the structure/function of wild-type proteins; DB: database; HEK: hyperechogenic kidneys; Mat: maternal inherited; P: pathogenic; VUS: variants of unknown significance; TOP: termination of pregnancy; Het: heterozygous; AD: autosomal dominant; LP: likely pathogenic; Pat: paternal inherited; AR: autosomal recessive; FGR: fetal growth restriction; PA: pulmonary atresia; HH5: Hypogonadotropic hypogonadism 5 with or without anosmia; KABUK1: Kabuki syndrome 1; AOS2: Adams-Oliver syndrome 2; NS1: Noonan syndrome 1; LPRD1: LEOPARD, syndrome 1.

### The detection rate of CMA

Of the 61 cases with clinically significant chromosomal abnormalities, 33 cases (7.1%) were identified with aneuploidies by QF-PCR and karyotype, including 15 cases of trisomy 21, 11 cases of trisomy 18, two cases of trisomy 13, three cases of Turner syndrome, one case of trisomy X, and one case of trisomy 22. Additionally, 28 (likely) pathogenic CNVs were detected by CMA (Supplementary Table S1). The increment of CMA in diagnosis following negative QF-PCR/karyotype increased by 6.4% (28/435). The most detected CNV is 22q11.2 microdeletion syndrome (8/28, 28.6%). VUS was found in 50/468 (10.68%) fetuses with VSDs.

### The detection rate of ES

In general, 51 fetuses with VSDs (12 isolated cases, 39 non-isolated cases) and negative CMA results received further Trio-WES detection. The incremental diagnostic rate of VSD fetuses by ES was 13.7% (7/51), including seven fetuses with (likely) pathogenic genetic variants. We detected nine fetuses harboring VUS (9/51, 17.6%) (Table 3). We detected seven (likely) pathogenic variants related to clinical phenotype in seven fetuses (Table 2), involving genes such as *NOTCH2*, *FANCI*, *CHD7*, *KMT2D*, *DOCK6*, *PTPN11*, etc. Six novel variants were not reported previously. Thus, in this study, we reported new variants in the VSD-related genes.

**TABLE 3:** Clinical characteristics of VSD fetuses with VUS.

Case	Ultrasound findings	Gene	Reference sequence	Chromosomal loci (GRCh37/hg19)	Nucleotide/Protein Position	Present in gnomAD/inhouse DB	Mutation type	Potential diagnosis	Outcome
1	VSD, FGR	CTU2	NM_001318507.1	chr16:88778594 chr16:88781053	c.469G>A (p. Glu157Lys) c.1473C>T (p. Pro491 = )	YES (0.0004427^#^)/YES (0.000199681*) NO/NO	Het, Pat, AR Het, Mat, AR	MFRG	TOP
2	VSD, Right-sided aortic arch	STAG2	NM_001042749.2	chrX:123224554	c.3407A>T (p. Asp1136Val)	NO/NO	Hemi, Mat, XL/XD/XR	MKMS; HPE13	TOP
3	Isolated VSD	HSPG2	NM_005529	chr1:22155942 chr1:22190714	c.11929G>A (p.V3976M) c.4627-8G>A	YES (0.0001^#^)/NO YES (0.00009^#^)/NO	Het, Mat, AR Het, Pat, AR	SJS1; DDSH	TOP
4	VSD, FGR	CHD4	NM_001273.5	chr12:6692288	c.4061–9T>C	NO/NO	Het, *De novo*, AD	SIHIWES	Neonatal death
5	VSD, Transposition of the great arteries	JAG1	NM_000214.3	chr20:10653546	c.190C>A (p. Arg64Ser)	NO/NO	Het, Mat, AD	ALGS1; TOF; CMT2HH	TOP
6	Isolated VSD	CDK8	NM_001260.2	chr13:26967652	c.790 + 5A>T	NO/NO	Het, *De novo*, AD	IDDHBA	Live birth
7	VSD, FGR	FBN1	NM_000138.4	chr15:48807661	c.1391G>A (p. Arg464His)	YES (0.000007960^#^)/NO	Het, Pat, AD	MFS; GPHYSD2	TOP
8	VSD, FGR	ROR2	NM_004560.3	chr9:94486564 chr9:94486659	c.2212C>T (p. Arg738Cys) c.2117G>A (p. Arg706Gln)	YES (0.002308^#^)/YES (0.004 *) YES (0.004364^#^)/NO	Het, Pat, AR/AD Het, Mat, AR/AD	RRS1; BDB1	TOP
9	PA, VSD	TRIO	NM_007118.3	chr5:14461226	c.5302C>T (p. Arg1768Trp)	NO/NO	Het, *De novo*, AD	MRD44	TOP

#: Allele frequencies of East Asian populations; *: Allele frequencies of Chinese populations; VUS: variants of unknown significance; VSD: ventricular septal defect; Het: heterozygous; AD: autosomal dominant; TOP: termination of pregnancy; Mat: maternal inherited; MFS: marfan syndrome; AR: autosomal recessive; Pat: paternal inherited; Hemi: hemizygous; XR: X-linked recessive; FGR: fetal growth restriction; MFRG: microcephaly, facial dysmorphism, renal agenesis, and ambiguous genitalia syndrome; MKMS: Mullegama-Klein-Martinez syndrome; HPE13: Holoprosencephaly 13, X-linked; SJS1: Schwartz-Jampel syndrome, type 1; DDSH: dyssegmental dysplasia, Silverman-Handmaker type; SIHIWES: Sifrim-Hitz-Weiss syndrome; ALGS1: Alagille syndrome 1; TOF: tetralogy of fallot; CMT2HH: Charcot-Marie-Tooth disease, axonal, type 2HH; IDDHBA: intellectual developmental disorder with hypotonia and behavioral abnormalities; MFS: marfan syndrome; GPHYSD2: Geleophysic dysplasia 2; RRS1: robinow syndrome, autosomal recessive; BDB1: brachydactyly, type B1; MRD44: Intellectual developmental disorder, autosomal dominant 44, with microcephaly.

Six variants were not previously reported, two variants were maternally and two were paternally inherited (Table 2). Among the seven (likely) pathogenic variants, three were missense variants, two were splice site variants, one was a frameshift variant, and one was a nonsense variant; the mode of transmission of the condition of the four novel variants was autosomal dominant (AD) inheritance. A novel variant c.4308delC (p. Ser1437ProfsTer69) in the *KMT2D* gene resulted in Kabuki syndrome type 1, KS (OMIM: 147920). The deletion of exons 2–4 in the *NOTCH2* gene may lead to HAJDU-CHENNEY syndrome (OMIM: 102500) and ALAGILLE syndrome type 2 (OMIM: 610205). The novel variant c.5464G > A (p. Gly1822Ser) in the *CHD7* gene can cause CHARGE syndrome (OMIM: 214800) and Hypogonadotropic hypogonadism 5 with or without anosmia, HH5 (OMIM: 612370). The mode of transmission of the condition of the remaining two variants was autosomal recessive (AR) (Two variants were found in the identified genes, see Table 2). Case 6 inherited the variation from their mother, c.3187–2A > G in the *FANCI* gene; case 8 inherited the variation from his father, c.807–1G > A in the *DOCK6* gene. The results of gene detection are presented in Table 2.

### Genetic diagnosis rate of isolated and non-isolated VSD

The non-isolated VSD group had a significantly higher detection rate of total (likely) pathogenic gene variations (7/133, 5.3% VS. 61/335, 18.2%, *p* = 0.000). Among them, the detection rates of CNVs (7.2% vs. 3.0%, *p* = 0.128) and sequence variants (15.4% vs. 8.3%, *p* = 1.000) in the non-isolated VSD group were higher than those in the isolated VSD group. The difference in the detection rate of fetal aneuploidy between the groups was significant (9.3% vs. 1.5%, *p* = 0.002), this is likely due to the inclusion of soft markers in the non-isolated group. Additionally, the detection rate of (likely) pathogenic variants in VSD patients with neurological abnormalities, skeletal abnormalities, and urinary system abnormalities was significantly higher than that of other types of extracardiac structural abnormalities (*p* < 0.05).

### Pregnancy outcomes

Perinatal outcomes were obtained in 451 cases (96.4%) (Table 4), of which 102 (21.8%) families chose to terminate a pregnancy, 344 (73.5%) families had live births, five (1.1%) newborns died after birth, and 17 (3.6%) cases were not followed up. The difference in the pregnancy termination rate and survival rate between the isolated and non-isolated VSD groups was significant (21.8% vs. 53.1% and 73.5% vs. 39.2%, *p* < 0.01). Among the 344 families who chose to continue their pregnancy, 121 families had normal post-natal examination results, of which isolated VSDs accounted for 50.4% (61/121). Among 121 families with normal *postpartum* examination results, 19 cases were false positives due to two-dimensional ultrasound echo loss artifacts and color Doppler overlap artifacts (however, we cannot rule out the possibility of VSD closure during the last trimester of gestation, as frequently happens.), 67 cases of muscular VSDs and 35 cases of other types of VSDs spontaneously closed *in utero*. This clinical data increased the confidence of families with fetuses diagnosed with VSDs to choose to continue the pregnancy.

**TABLE 4:** Statistical analysis of the genetic and clinical outcomes of VSD fetuses.

Groups	QF-PCR	CMA			ES					
	Aneuploidies	P/LP (CNVs)	VUS	Live birth	Normal post-natal exam	Groups	P/LP	VUS	Live birth	Normal post-natal exam
**Isolated vs. non-isolated**						**Isolated vs. non-isolated**				
Isolated (n = 133)	2 (1.5%)	4 (3.0%)	9 (6.8%)	102 (76.7%)	34 (33.3%)	Isolated (n = 12)	1 (8.3%)	2 (16.6%)	10 (83.3%)	1(10.0%)
Non-isolated (n = 335)	31(9.3%)	24 (7.2%)	41 (12.2%)	242 (72.2%)	31(12.8%)	Non-isolated (n = 39)	6 (15.4%)	7 (17.9%)	20 (51.3%)	4 (20.0%)
*p*-value	0.002	0.128	0.084	0.325	0.000	*p*-value	1.00	1.00	0.049	0.640

VSD, ventricular septal defect; CMA, chromosomal microarray analysis; ES, exome sequencing; P, pathogenic; LP, likely pathogenic; VUS, variants of unknown significance.

## Discussion

Ventricular septal defect (VSD) is a very common congenital structural abnormality (Ferencz et al., 1987). Previous studies on the genetic etiology of fetuses with CHDs, including CMA and ES, were conducted with only a few individuals, and diagnostic rates ranged from 2.6% to 23.1% (Yates et al., 2017; Fu et al., 2018; Hu et al., 2018; Lord et al., 2019; Petrovski et al., 2019; Westphal et al., 2019; Sun et al., 2020; Mastromoro et al., 2022). In this study, we described the detection rate of different molecular diagnostic techniques for fetal VSD genetic causes and compared the genetic variation rates of isolated VSD and non-isolated VSD fetuses. This was the largest prenatal study on VSD yet. There were 61 cases (13.03%, 61/468) of chromosomal abnormalities and eight cases (13.7%, 7/51) of sequence variations. In a meta-analysis, Mastromoro et al., 2022. proposed that the incremental CMA diagnostic yield of VSD in fetuses with isolated cardiovascular malformations was the lowest, only 2.64%. The detection rate of our study was 13.03%, higher than that of them, which may be related to sample selection bias. After excluding 33 cases of chromosome aneuploidies, the total incidence of CNVs (28/468) and sequence variations (7/51) was 7.5% (35/468), which was lower than that in a recent study (15.7%) (van Nisselrooij et al., 2020). The reason for the lower diagnosis rate than that in the above study might be that only 51 families with negative CMA results in our center opted for ES, which was considerably lower than the number of cases in the study conducted by Van et al. However, the (likely) pathogenic variants were detected in 13.7% (7/51) of fetal VSD pregnancies with negative karyotype and CMA results, which indicated that ES might have a high additional diagnostic rate for molecular diagnosis of fetal VSD. In our study, we identified six novel variants and expanded variants spectra of VSD-related genes. Furthermore, We summarize the literature on the application of CMA/ES in the prenatal diagnosis of fetal congenital heart disease in Table 5 and Table 6.

**TABLE 5:** Summary of the literature on the application of CMA in prenatal diagnosis of fetal congenital heart disease (n ≥ 100).

Source of research	Inclusion criteria of cases	Isolated group		Non-isolated group			Total		
		Number of cases (n)	Diagnostic rate (n%)	Number of cases (n)	Diagnostic rate (n%)	Number of cases (n)	Diagnostic rate of aneuploidy (n%)	Diagnostic rate of CNV (n%)	Total diagnostic rate (n%)
Lu et al., 2022	All types of CHD	134	20.9	66	31.8	200	11.5	13.0	24.5
Zhang et al., 2022	All types of CHD	867	8.1	168	21.4	1035	4.8	5.3	10.1
Qiao et al., 2021	All types of CHD	277	12.3	83	31.3	360	8.1	8.6	16.7
Sagi-Dain et al. 201	All types of CHD	1433	3.8	295	13.2	1728	3.1	2.3	5.4
Mustafa et al., 2020	All types of CHD	141	22.0	76	64.5	217	29.5	7.4	36.9
Song et al., 2019	All types of CHD	123	9.8	67	9.0	207	8.2	8.7	16.9
Turan et al., 2018	All types of CHD	92	16.4	53	24.5	145	14.0	20.0	34.0
Wang et al., 2018	All types of CHD	421	14.3	181	35.9	602	14.1	14.3	28.4
Hureaux et al., 2019	Isolated CHD	239	7.9	NA	NA	239	NA	7.9	7.9
Maya et al., 2020	VSD	568	1.4	123	14.6	691	2.0	1.7	3.8
Cai et al., 2018	VSD	79	1.3	72	36.1	151	9.3	13.3	22.5
Fu et al., 2017	VSD	73	5.5	71	11.3	144	NA	8.3	8.3
Cheng et al., 2022	Isolated VSD	168	4.2	NA	NA	168	NA	4.2	4.2

CHD, congenital heart defect.

**TABLE 6:** Summary of the literature on the application of ES in prenatal diagnosis of fetal congenital heart disease (n ≥ 50).

Source of research	Inclusion criteria of cases	Isolated group		Non-isolated group		Total	
		Number of cases (n)	Diagnostic rate (n%)	Number of cases (n)	Diagnostic rate (n%)	Number of cases (n)	Total diagnostic rate (n%)
Lu et al., 2022	All types of CHD	44	9.1	8	25.0	52	11.5
Chen et al., 2022	All types of CHD	NA	NA	NA	NA	235	16.2
Qiao et al., 2021	All types of CHD	250	7.2	50	12.0	300	8.0
Mone et al., 2021	All types of CHD	122	11.5	75	14.7	197	12.7
Li et al., 2020	All types of CHD	190	7.9	70	15.7	260	10.0
Lord et al., 2019	All types of CHD	NA	NA	NA	NA	81	11.1
Petrovski et al., 2019	All types of CHD	49	2.0	28	10.7	77	5.1
Yi et al., 2022	Visceral inversion	NA	NA	NA	NA	69	11.6
Sun et al., 2020	Left heart abnormality	53	15.1	13	38.5	66	19.7

CHD, congenital heart defect Declarations.

We found that the detection rate of total (likely) pathogenic gene variants in the non-isolated VSD group (61/335, 18.2%) was significantly higher than that in the isolated VSD group (7/133, 5.3%) (*p* < 0.05). In addition, the difference in the aneuploidy detection rates between the two groups was significant (9.3% vs. 1.5%, *p* = 0.002). The difference in incidence of CNVs and sequence variations between the groups was significantly different, which indicated that VSD was more likely to be related to chromosomal abnormalities and sequence variations when occurring with other structural abnormalities. This finding matched the conclusions of other studies but did not match the findings of Qiao et al., 2021. (van Nisselrooij et al., 2020; Mone et al., 2021). The disagreement with the findings of Qiao et al., 2021. might be because our study was limited to prenatal VSD fetuses, while the study conducted by Qiao et al. included various subtypes of CHD fetuses. These inconsistent findings might also be partially explained by the distribution of CHD subtypes, the proportion of isolated VSDs detected in the current cohort, and the differences in the detection methods used. We suggest that clinicians should consider a wider range of differential diagnoses for non-isolated VSD, including the diagnosis of more multi-system syndromes. These reliable phenotypes can facilitate the analysis of ES and the best practices recommend both a phenotype and genotype driven analysis approach (Austin-Tse et al., 2022; Fu et al., 2022). Considering VSD has a high detection rate (isolated or non-isolated), we suggest invasive prenatal diagnosis and genomic analysis for all ultrasound-detected VSDs. However, the classification of non-isolated and isolated VSD depends on the results of prenatal ultrasound examinations. In the prenatal environment, determining a complete and accurate fetal phenotype is not always possible due to different manifestations of fetal disease, incomplete penetrance, and the presence of ultrasound abnormalities only in late pregnancy. Additionally, some phenotypes, such as intellectual disability, developmental delay, minor malformations, or metabolic abnormalities, cannot be detected by prenatal imaging. These restrictions may increase the complexity and difficulty of prenatal ES analysis (Li et al., 2020).

In this study, we identified six novel variants associated with the prenatal phenotype of VSD for the first time and expanded the variant spectrum of VSD-related genes. Sun et al. conducted a study on prenatal cardiac left-sided lesions and found that 19.7% (13/66) of cases had diagnostic variants, and 10.6% (7/66) of cases had *KMT2D* variants (Sun et al., 2020). However, in this study, only one fetus had a new truncated variant in the *KMT2D* gene. *KMT2D* variants can affect the function of H3K4-ASCOM, which can lead to estrogen receptor-mediated pathway disorders and cause a series of KS (OMIM: 147920) phenotypes, including VSD (Miyake et al., 2013). Considering that KS is not rare, the penetrance of patients with *KMT2D* pathogenic variants is complete. About 70% of KS patients have congenital heart defects and risk of intellectual disability (Digilio et al., 2017). Therefore, we suggest that the variation of the *KMT2D* gene should be considered in the prenatal diagnosis of Chinese pregnant women with suspected VSD or other congenital facial abnormalities. The clinical management of *KMT2D* gene variants should include echocardiography at diagnosis for early diagnosis and treatment. Our study expanded the variant spectrum of the *KMT2D* gene, and our findings might contribute to genetic counseling.

Some genes and genetic pathways are related to heart development, and the abnormalities of these genes are related to the form and function of congenital heart diseases (Afouda et al., 2008; Aoki et al., 2008; McCulley and Black, 2012). In this study, we detected one variant in the TRIO gene (c.5302C, 181 > T p. Arg1768Trp) in case 5. At present, there is no research has been reported to be associated with VSD or other CHDs. Although it is possible this is a new disease gene, however, a more likely explanation is that this is an incidental finding in this case and is unrelated to the VSD presentation. Two novel variants of the *PTPN11* gene, (c.1507G>A, p. Gly503Arg) and (c.836A>G, p. Tyr279Cys), were identified. Both were missense variants located in the protein tyrosine phosphatase, catalytic domain (PTPcs) encoded by the *PTPN11* gene. The bioinformatics software predicted that these two variant loci might disrupt wild-type protein structure/function. This variant (c.836A>G, p. Tyr279Cys) was included in the ClinVar database as pathogenic or likely pathogenic (Variation ID: 13328, two stars) and included in HGMD with ID CM021133. Some functional experimental studies showed that this variant could disrupt the enzymatic activity of *PTPN11 in vitro* and increase the activity of AKT and mTOR in cell culture (Hanna et al., 2006; Kontaridis et al., 2006; Martinelli et al., 2008; Marin et al., 2011; Schramm et al., 2013). This variant (c.1507G>A, p. Gly503Arg) was included in the dbSNP database with the ID rs397507545 but not in the 1000 Genomes and gnomAD databases. The variant locus was included in the ClinVar database as pathogenic (Variation ID: 40559, two stars); it was included in HGMD with ID CM060440. *PTPN11* is a cytoplasmic tyrosine phosphatase involved in signaling pathways that are induced by growth factors, cytokines, hormones, and extracellular matrix. It regulates the RAS/mitogen-activated protein kinase (MAPK) pathway. Approximately 50% of Noonan syndrome is caused by functionally acquired missense variants in the *PTPN11* gene (Adam et al., 1993). These variants are mainly in the N-SH2 or PTP structural domain, which prevents the inhibition of the N-SH2 structural domain. The PTP structural domain is also activated in the absence of phosphorylated ligand binding and upregulates the Ras/MAPK signaling pathway, leading to the development of Noonan syndrome. The pathogenesis of Noonan syndrome, LEOPARD syndrome, Costello syndrome, and cardiofacial syndrome all involve the Ras/MAPK signaling pathway. Recent studies have also found that many genes are involved in the morphogenesis of the cardiac septum and the development of the cardiac cavity. Our findings suggested that patients with prenatal suspicion of CHDs should undergo routine screening for variants in the *PTPN11* gene. However, it is undeniable that the overlapping features observed in RASopathies, the wide range of phenotypes within each trait, and the lack of clinical features with pathological diagnostic value, as well as consensus on specific and routinely used diagnostic criteria, make the prenatal diagnosis and genetic counseling of RASopathies challenging.

It is worth noting that in our study, pregnant women (7/9, 77.8%) with genetic variations classified as VUS chose to terminate the pregnancy. According to the follow-up, the main reasons why pregnant women choose to terminate pregnancy include the uncertainty of fetal prognosis, emotional factors, and family economic factors. This issue triggers our thinking as follows. When prenatal diagnosis results are classified as VUS, clinicians and genetic counselors should pay special attention to whether they are from parents or *de novo*. Clinicians and genetic counselors should find out as much current information as possible to provide the most adequate and detailed genetic counseling for both couples in order to make the best clinical decision and reduce the rate of blind termination of pregnancy. The post-natal follow-up of children with a prenatal diagnosis of fetal VSD is necessary. We collected data on pregnancy outcomes and *postpartum* treatment of these live birth VSD cases described in this study. Our findings showed that isolated VSDs had a better prenatal and post-natal prognosis than non-isolated VSDs, and the combination of fetal VSDs with other abnormalities was associated with increased rates of pregnancy termination and post-natal surgery. We also found that the detection rate of total (likely) pathogenic genetic variants was significantly higher in the non-isolated VSD group (61/335, 18.2%) than in the isolated group (7/133, 5.3%) (*p* < 0.05). Overall, a definitive genetic diagnosis and a good prognosis can help in increasing the confidence of patients to continue their pregnancy, especially those carrying an isolated VSD fetus with a negative genetic test result.

The advantage of this study is that we performed genetic diagnosis in a large cohort of unselected VSD fetuses without sample bias and therefore were able to assess reliable incremental yield. This study also had low heterogeneity. However, our study had several limitations. First, this was a retrospective study with possible recall bias, and obtaining fresh blood from patients for cell or protein chemistry experiments was difficult. Second, we might have overlooked balanced chromosomal rearrangements, low-ratio mosaics, and variants in regulatory elements, intergenic regions, and untranslated regions. Third, in our study, for patients with isolated VSD, there was an ES diagnostic rate of 1/12 (0.08%, 95% CI 0.01–0.35), this is vastly insufficient evidence to determine whether ES is warranted. Larger studies are required to determine the diagnostic yield in this group in the future. Fourth, VUS identified by ES, especially in proband-only samples, might have complicated prenatal counseling and parental decision-making. However, these variations will eventually be characterized and reduced as more studies are conducted. In addition, as this study is a retrospective review of the results of CMA and ES testing performed over the course of 10 years, we cannot rule out the impact of any evolution of technology used during the period of study on the testing results.

## Conclusion

In our study, CNVs and gene sequence variations were found in many cases with VSD. We suggest that pregnant women carrying fetuses with VSDs combined with additional associated anomalies, but without chromosomal abnormalities and CNVs, can undergo ES. However, the effectiveness of ES in isolated VSD fetuses needs to be confirmed by larger studies in the future.

## Data Availability

The datasets presented in this study can be found in online repositories. The names of the repository/ repositories and accession number(s) can be found in the article/Supplementary Material.
