# Supplementary material for: Prenatal genetic diagnosis associated with fetal ventricular septal defect: an assessment based on chromosomal microarray analysis and exome sequencing
Source: Front Genet. 2023 Nov 24;14:1260995. doi: 10.3389/fgene.2023.1260995 (PMC10704506; doi:10.3389/fgene.2023.1260995)
Supplement: Supplementary file 1 [file Table8.DOCX]

**
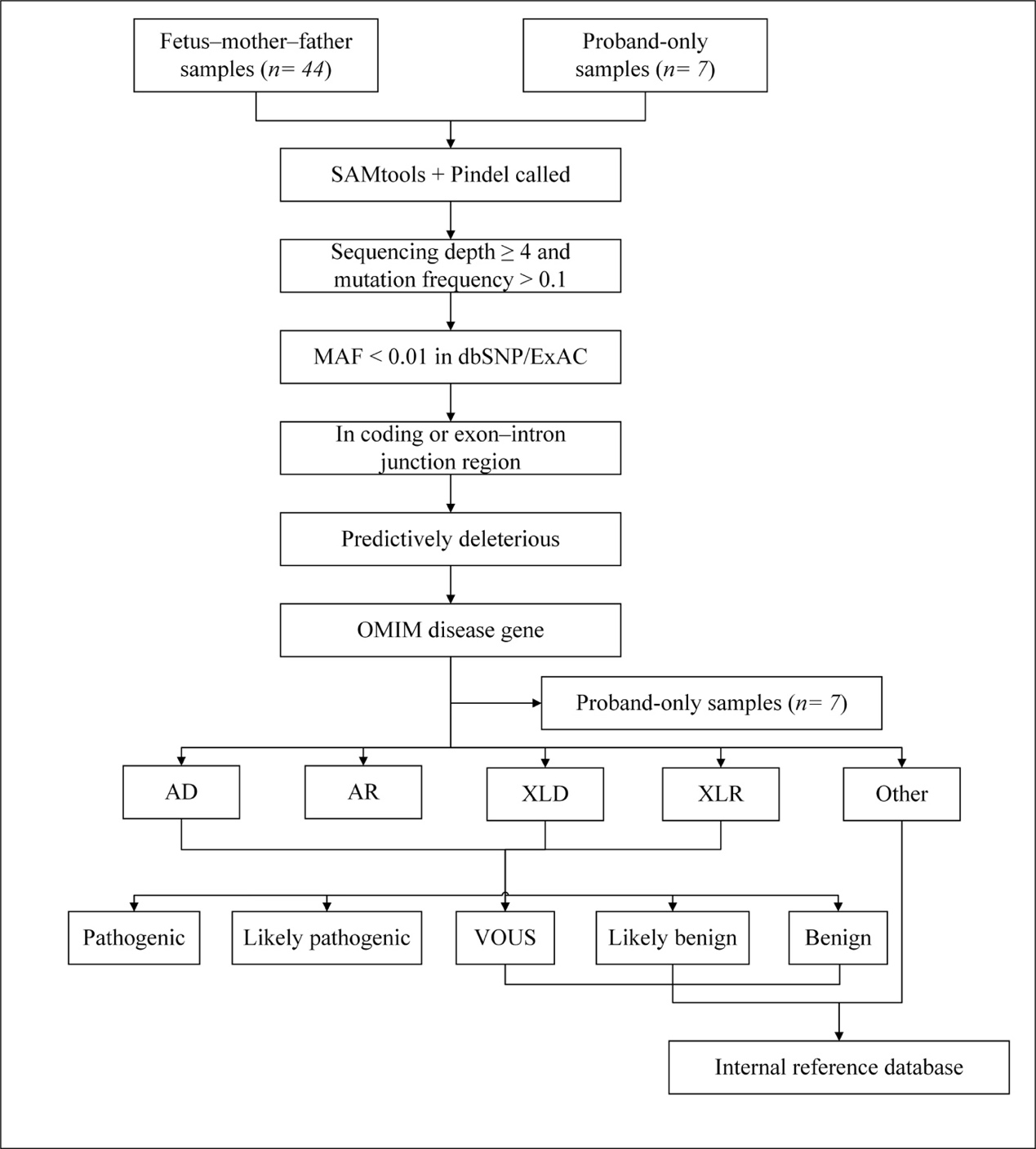
 Fig. S1** Flowchart of whole exome sequencing data analysis process in fetuses with VSD. 5. AD, autosomal dominant; AR, autosomal recessive; dbSNP, SNP database; ExAC, Exome Aggregation Consortium database; MAF, minor allele frequencies; OMIM, Online Mendelian Inheritance in Man database; SAM, sequence alignment/map; VOUS, variants of unknown signifificance; XLD, X-linked dominant; XLR, X-linked recessive.
