# Supplementary material for: Prenatal genetic diagnosis associated with fetal ventricular septal defect: an assessment based on chromosomal microarray analysis and exome sequencing
Source: Front Genet. 2023 Nov 24;14:1260995. doi: 10.3389/fgene.2023.1260995 (PMC10704506; doi:10.3389/fgene.2023.1260995)
Supplement: Supplementary file 2 [file Table7.DOCX]

**Supplementary file 1.** Analysis and interpretation process of ES data

**Exome sequencing (ES)**

Fetuses with negative karyotyping and CMA results and their parents were examined for Trio-ES. Only cases that obtained parental consent and preserved sufficient and qualified DNA samples could receive Trio-ES. With signed written informed consent, we performed targeted enrichment of the DNA samples using the Agilent SureSelect human exome capture probes (V6, Life Technologies, Carlsbad, CA, USA) according to the manufacturer's protocol. The DNA library was sequenced using Illumina HiSeq2500, HiSeq Xten, or NovaSeq platforms to obtain 150 bp paired-end reads.

Raw fastq reads were filtered by using Trimmomatic(Bolger, Lohse, and Usadel 2014) (v0.36) or fastp (Chen et al. 2018) (v0.20/v0.23) to remove low quality and adapter contaminated reads, leaving clean reads aligned to the human reference genome (GRCh37/Hg19) with BWA (Li and Durbin 2009) (v0.7.17) mem algorithm, with Samtools (Li et al. 2009) (v1.3.1/v1.9) and Picard (v2.17.1) converted to BAM format and PCR duplicates were discarded. Genome Analysis ToolKit (McKenna et al. 2010) (GATK v3.6/v3.8) was employed for local indel realignment, base quality recalibration and haplotypecaller variant calling. Variant annotation was conducted with Ensembl’s Variant Effect Predictor (McLaren et al. 2016) (VEP v85/v104) and Annovar (Wang, Li, and Hakonarson 2010) (v2017Jul17/v2020Apr01). Allele frequency information from 1000 Genome Project (1000G Phase 3 v5a), Genome Aggregation Database (gnomAD r2.1/v2.1.1), Exome Aggregation Consortium (ExAC r0.3.1) and the Exome Sequencing Project (ESP v2) were annotated. Multiple software such as SIFT (Sim et al. 2012), Polyphen2 (Adzhubei et al. 2010), MutationTaster (Schwarz et al. 2014), MutationAssessor (Reva, Antipin, and Sander 2011), Provean (Choi and Chan 2015), CADD (Kircher et al. 2014) and REVEL (Ioannidis et al. 2016) were applied for protein function prediction and Human Splicing Finder (Desmet et al. 2009), MaxEntScan (Yeo and Burge 2004), NNSplice (Reese et al. 1997), GeneSplicer (Pertea, Lin, and Salzberg 2001) and SpliceAI (Jaganathan et al. 2019) were performed to assess potential impacts on splicing. Gene/variants were additionally annotated according to ClinVar, ClinGen, the professional version of the Human Gene Mutation Database (HGMD professional v2018.2 & v2021.2), previously associated diseases (based on Online Mendelian Inheritance in Man and Orphanet), and known functional domain data (according to UniProtKB and Human Protein Reference Database). Besides, the imprinted gene (Geneimprint and MetaImprint database) and genome segmental duplication region (downloaded from the UCSC genome browser) were added to the VCF file annotation. Two aspects were evaluated for gender determination, average depth of specific genes on chromosome Y and the heterozygous variants percentage on chromosome X. Then KING (Manichaikul et al. 2010) and PLINK (Chang et al. 2015) were used to confirm the family pedigree relationship.

Quality control for each sample included an average depth of > 60X and > 90% targeted region with at least 20X in this retrospective study. Variants with poor quality were discarded if meeting one of the following criteria, (1) with a depth (DP) <5X; (2) alternate allele proportion (AAP) <0.25; (3) mapping quality (MQ) <40; (4) genotype quality (GQ) <50. All the annotated variants, excluding low quality ones were subject to downstream analysis with the in-house script. Variants with a minor allele frequency (MAF) >5% were filtered out except for those in HGMD, ClinVar and ClinGen BA1 exception list (Ghosh et al. 2018) (BA1). Next, we mainly focused on genomic regions known or likely associated with the disease. Based on VEP functional consequence, potential protein-altering variants (e.g., missense, start loss, stop gain/loss, frameshift, in-frame insertion/deletion, or canonical splice-site) were retained. To aid data interpretation, major indications for ES for each fetus were extracted from clinical notes and converted into the standard Human Phenotype Ontology (HPO) terms.

A genotype-driven short rare variant list was prioritized for each trio with the help of local population data (more than 10,000 individuals including both patients and healthy individuals), (1) dominant *de novo* variants; (2) recessive homozygous variants (no homozygotes in the gnomAD2.1 and internal healthy controls); (3) recessive compound heterozygous variants; (4) *De novo* X chromosome variants or rare hemizygous variants inherited from mother; (5) known disease-causing alleles (ClinVar 3- or 4- star variants); (6) predicted truncating variants (nonsense, frameshift, canonical splice sites) with extremely low allele frequency (<0.01%). This short gene/variant list was then reviewed for clinical correlation and potentially relevant variants were classified based on the American College of Medical Genetics and Genomics (ACMG) guideline [23] and ClinGen VCEP gene-specific criteria [24-31] (when applicable). In addition, *bona fide* disease-causing variants (unrelated to fetal phenotype) with zygosity consistent with disease mode of inheritance in ACMG SF2.0 and childhood-onset disease genes were categorized as potential secondary or incidental findings.

Next, for unsolved cases without a clear answer to the clinical question, a comprehensive review of all rare variants in genes potentially related to clinical indications for prenatal diagnosis was performed with the aid of HPO matching. A gene was considered associated with the fetal anomalies meeting one of the following conditions, the clinical phenotypes of the disease gene should: (1) match HPO entry of the fetal phenotype; (2) match the superclass based on HPO or clinical synopsis in OMIM database; (3) be reported in previous cases manifesting the same or similar phenotypes of the fetuses.

In both steps, ES results were classified into five tiers: (1) positive diagnostic result: P/LP variants identified in a disease gene that can interpret (partly or fully) the fetal phenotype; (2) inconclusive: variant of unknown significance (VUS) identified in a disease gene which can explain (partly or fully) the fetal phenotype; (3) incidental findings (IFs): P/LP variants identified in childhood-onset disease gene, unrelated to fetal phenotype; (4) secondary findings (SFs): P/LP variants identified in genes unrelated to fetal phenotype, according to ACMG recommended list (Miller et al. 2021; Kalia et al. 2017); (5) candidate genes: variants (primarily *de novo*) predicted to be deleterious and absent in general population, identified in undefined disease genes that have a paralog gene or previously published data to support the association with fetal anomalies, or based on animal model and tissue expression.

The ES report included positive diagnostic and inconclusive results related to primary prenatal indications. Incidental and secondary findings with a childhood-onset disease were also included in the report, based on consensus between laboratory and clinicians. Secondary findings with a late-onset disease were not routinely reported.

**Tools:**

Trimmomatic: http://www.usadellab.org/cms/?page=trimmomatic

Fastp: https://github.com/OpenGene/fastp

BWA: https://bio-bwa.sourceforge.net/

Samtools: https://samtools.sourceforge.net/

Picard: https://broadinstitute.github.io/picard/

Genome Analysis ToolKit: https://gatk.broadinstitute.org/hc/en-us

Ensembl’s Variant Effect Predictor: https://www.ensembl.org/info/docs/tools/vep/index.html

Annovar: https://annovar.openbioinformatics.org/en/latest/

SIFT: https://sift.bii.a-star.edu.sg/

Polyphen2: http://genetics.bwh.harvard.edu/pph2/

MutationTaster: https://www.mutationtaster.org/

MutationAssessor: http://mutationassessor.org/r3/

Provean: https://www.jcvi.org/research/provean

CADD: https://cadd.gs.washington.edu/

REVEL: https://sites.google.com/site/revelgenomics/

Human Splicing Finder: http://www.umd.be/HSF3/HSF.shtml

MaxEntScan: http://hollywood.mit.edu/burgelab/maxent/Xmaxentscan_scoreseq.html

NNSplice: http://www.fruitfly.org/seq_tools/splice.html

GeneSplicer: https://ccb.jhu.edu/software/genesplicer/

SpliceAI: https://github.com/Illumina/SpliceAI

KING: https://www.kingrelatedness.com/manual.shtml

PLINK: https://www.cog-genomics.org/plink/

**References**

Adzhubei, I. A., S. Schmidt, L. Peshkin, V. E. Ramensky, A. Gerasimova, P. Bork, A. S. Kondrashov, and S. R. Sunyaev. 2010. 'A method and server for predicting damaging missense mutations', *Nat Methods*, 7: 248-9.

Bolger, A. M., M. Lohse, and B. Usadel. 2014. 'Trimmomatic: a flexible trimmer for Illumina sequence data', *Bioinformatics*, 30: 2114-20.

Chang, C. C., C. C. Chow, L. C. Tellier, S. Vattikuti, S. M. Purcell, and J. J. Lee. 2015. 'Second-generation PLINK: rising to the challenge of larger and richer datasets', *Gigascience*, 4: 7.

Chen, S., Y. Zhou, Y. Chen, and J. Gu. 2018. 'fastp: an ultra-fast all-in-one FASTQ preprocessor', *Bioinformatics*, 34: i884-i90.

Choi, Y., and A. P. Chan. 2015. 'PROVEAN web server: a tool to predict the functional effect of amino acid substitutions and indels', *Bioinformatics*, 31: 2745-7.

Desmet, F. O., D. Hamroun, M. Lalande, G. Collod-Beroud, M. Claustres, and C. Beroud. 2009. 'Human Splicing Finder: an online bioinformatics tool to predict splicing signals', *Nucleic Acids Res*, 37: e67.

Ghosh, R., S. M. Harrison, H. L. Rehm, S. E. Plon, L. G. Biesecker, and Group ClinGen Sequence Variant Interpretation Working. 2018. 'Updated recommendation for the benign stand-alone ACMG/AMP criterion', *Hum Mutat*, 39: 1525-30.

Ioannidis, N. M., J. H. Rothstein, V. Pejaver, S. Middha, S. K. McDonnell, S. Baheti, A. Musolf, Q. Li, E. Holzinger, D. Karyadi, L. A. Cannon-Albright, C. C. Teerlink, J. L. Stanford, W. B. Isaacs, J. Xu, K. A. Cooney, E. M. Lange, J. Schleutker, J. D. Carpten, I. J. Powell, O. Cussenot, G. Cancel-Tassin, G. G. Giles, R. J. MacInnis, C. Maier, C. L. Hsieh, F. Wiklund, W. J. Catalona, W. D. Foulkes, D. Mandal, R. A. Eeles, Z. Kote-Jarai, C. D. Bustamante, D. J. Schaid, T. Hastie, E. A. Ostrander, J. E. Bailey-Wilson, P. Radivojac, S. N. Thibodeau, A. S. Whittemore, and W. Sieh. 2016. 'REVEL: An Ensemble Method for Predicting the Pathogenicity of Rare Missense Variants', *Am J Hum Genet*, 99: 877-85.

Jaganathan, K., S. Kyriazopoulou Panagiotopoulou, J. F. McRae, S. F. Darbandi, D. Knowles, Y. I. Li, J. A. Kosmicki, J. Arbelaez, W. Cui, G. B. Schwartz, E. D. Chow, E. Kanterakis, H. Gao, A. Kia, S. Batzoglou, S. J. Sanders, and K. K. Farh. 2019. 'Predicting Splicing from Primary Sequence with Deep Learning', *Cell*, 176: 535-48 e24.

Kalia, S. S., K. Adelman, S. J. Bale, W. K. Chung, C. Eng, J. P. Evans, G. E. Herman, S. B. Hufnagel, T. E. Klein, B. R. Korf, K. D. McKelvey, K. E. Ormond, C. S. Richards, C. N. Vlangos, M. Watson, C. L. Martin, and D. T. Miller. 2017. 'Recommendations for reporting of secondary findings in clinical exome and genome sequencing, 2016 update (ACMG SF v2.0): a policy statement of the American College of Medical Genetics and Genomics', *Genet Med*, 19: 249-55.

Kircher, M., D. M. Witten, P. Jain, B. J. O'Roak, G. M. Cooper, and J. Shendure. 2014. 'A general framework for estimating the relative pathogenicity of human genetic variants', *Nat Genet*, 46: 310-5.

Li, H., and R. Durbin. 2009. 'Fast and accurate short read alignment with Burrows-Wheeler transform', *Bioinformatics*, 25: 1754-60.

Li, H., B. Handsaker, A. Wysoker, T. Fennell, J. Ruan, N. Homer, G. Marth, G. Abecasis, R. Durbin, and Subgroup Genome Project Data Processing. 2009. 'The Sequence Alignment/Map format and SAMtools', *Bioinformatics*, 25: 2078-9.

Manichaikul, A., J. C. Mychaleckyj, S. S. Rich, K. Daly, M. Sale, and W. M. Chen. 2010. 'Robust relationship inference in genome-wide association studies', *Bioinformatics*, 26: 2867-73.

McKenna, A., M. Hanna, E. Banks, A. Sivachenko, K. Cibulskis, A. Kernytsky, K. Garimella, D. Altshuler, S. Gabriel, M. Daly, and M. A. DePristo. 2010. 'The Genome Analysis Toolkit: a MapReduce framework for analyzing next-generation DNA sequencing data', *Genome Res*, 20: 1297-303.

McLaren, W., L. Gil, S. E. Hunt, H. S. Riat, G. R. Ritchie, A. Thormann, P. Flicek, and F. Cunningham. 2016. 'The Ensembl Variant Effect Predictor', *Genome Biol*, 17: 122.

Miller, D. T., K. Lee, W. K. Chung, A. S. Gordon, G. E. Herman, T. E. Klein, D. R. Stewart, L. M. Amendola, K. Adelman, S. J. Bale, M. H. Gollob, S. M. Harrison, R. E. Hershberger, K. McKelvey, C. S. Richards, C. N. Vlangos, M. S. Watson, C. L. Martin, and Acmg Secondary Findings Working Group. 2021. 'ACMG SF v3.0 list for reporting of secondary findings in clinical exome and genome sequencing: a policy statement of the American College of Medical Genetics and Genomics (ACMG)', *Genet Med*, 23: 1381-90.

Pertea, M., X. Lin, and S. L. Salzberg. 2001. 'GeneSplicer: a new computational method for splice site prediction', *Nucleic Acids Res*, 29: 1185-90.

Reese, M. G., F. H. Eeckman, D. Kulp, and D. Haussler. 1997. 'Improved splice site detection in Genie', *J Comput Biol*, 4: 311-23.

Reva, B., Y. Antipin, and C. Sander. 2011. 'Predicting the functional impact of protein mutations: application to cancer genomics', *Nucleic Acids Res*, 39: e118.

Schwarz, J. M., D. N. Cooper, M. Schuelke, and D. Seelow. 2014. 'MutationTaster2: mutation prediction for the deep-sequencing age', *Nat Methods*, 11: 361-2.

Sim, N. L., P. Kumar, J. Hu, S. Henikoff, G. Schneider, and P. C. Ng. 2012. 'SIFT web server: predicting effects of amino acid substitutions on proteins', *Nucleic Acids Res*, 40: W452-7.

Wang, K., M. Li, and H. Hakonarson. 2010. 'ANNOVAR: functional annotation of genetic variants from high-throughput sequencing data', *Nucleic Acids Res*, 38: e164.

Yeo, G., and C. B. Burge. 2004. 'Maximum entropy modeling of short sequence motifs with applications to RNA splicing signals', *J Comput Biol*, 11: 377-94.
