# Supplementary material for: Prenatal genetic diagnosis associated with fetal ventricular septal defect: an assessment based on chromosomal microarray analysis and exome sequencing
Source: Front Genet. 2023 Nov 24;14:1260995. doi: 10.3389/fgene.2023.1260995 (PMC10704506; doi:10.3389/fgene.2023.1260995)
Supplement: Supplementary file 3 [file Table9.DOCX]

**Table S1** Clinically relevant characteristics of VSD fetuses and clinically significant CMA findings.

| **Case** | **MA (years)** | **GA (weeks)** | **Ultrasound findings** | **CMA findings** | **Type of CNV** | **Size (Mb)** | **Interpretation** | **Outcomes** |
| --- | --- | --- | --- | --- | --- | --- | --- | --- |
| 1 | 33.3 | 24.6 | Isolated VSD | arr16p13.13p13.12(12,512,660-14,514,019) ×1 | Deletion | 3.20 | LP | Live birth |
| 2 | 32.1 | 24.0 | VSD, HEK | arr22q11.21q11.23(21465661-23810042) ×1 | Deletion | 2.34 | P | TOP |
| 3 | 26.3 | 30.7 | VSD, HEK | arr22q11.21(18916842-21800797) ×1 | Deletion | 2.88 | P | TOP |
| 4 | 42.8 | 20.3 | Complete VSD, AVSD | arr2q24.1q24.3(155838041-168073532) ×1 | Deletion | 12.24 | P | TOP |
| 5 | 37.6 | 22.3 | VSD, aortic arch hypoplasia | arr22q11.21(18916842-21798907) ×1 | Deletion | 2.88 | P | TOP |
| 6 | 29.4 | 29.6 | VSD, unilateral foot varus | arr4p16.3p15.33(68345-14195870) ×1 | Deletion | 14.13 | P | TOP |
| 7 | 29.1 | 24.9 | VSD, oligohydramnios | arr9p24.3p11.2(258491-44900526) ×3  arr9q13q21.32(66837485-86009559) ×3 | Duplication  Duplication | 44.64  19.17 | P  P | TOP |
| 8 | 23.2 | 29.4 | VSD, FGR | arr9q34.3(138311547-141020389) ×3  arr12q24.32q24.33(126394843-133777902) ×1 | Duplication Deletion | 2.71  7.38 | P  P | TOP |
| 9 | 29.9 | 26.0 | VSD, aortic arch hypoplasia | arr4p16.3p15.33(68345-13604913) ×1 | Deletion | 13.54 | P | TOP |
| 10 | 26.8 | 23.3 | Isolated VSD | arr22q11.21(18644790-21465662) ×1 | Deletion | 2.82 | P | TOP |
| 11 | 28.6 | 25.7 | VSD, AVSD | arr7q11.23(72718277-74142190) ×1 | Deletion | 1.42 | P | Live birth |
| 12 | 23.2 | 25.3 | Isolated VSD | arr12q15q21.1(69281650-73389759) ×1 | Deletion | 4.11 | LP | TOP |
| 13 | 30.6 | 24.7 | VSD, HEK | arr22q11.21(18644790-21465662) ×1 | Deletion | 2.82 | P | TOP |
| 14 | 28.7 | 24.7 | VSD, aortic arch hypoplasia | arr22q11.21(18631365-21800471) ×1 | Deletion | 3.71 | P | TOP |
| 15 | 31.9 | 23.4 | VSD, tricuspid regurgitation | arr16p11.2(29412503-30190029) ×1 | Deletion | 0.778 | P | TOP |
| 16 | 28.2 | 25.6 | VSD, PA | arr10q23.1q26.3(86,892,011-135,426,386) ×3 | Duplication | 48.53 | P | TOP |
| 17 | 31.1 | 25.0 | VSD, HEK | arr22q11.21(18645353-21798907) ×1 | Deletion | 3.15 | P | TOP |
| 18 | 28.8 | 32.6 | VSD, aortic arch hypoplasia | arr22q11.21(18916842-21465659) ×1 | Deletion | 2.55 | P | TOP |
| 19 | 23.4 | 26.7 | VSD, Echogenic intracardiac focus | arr22q11.21q11.23(21465661_23810042) ×1 | Deletion | 2.34 | P | TOP |
| 20 | 34.9 | 23.9 | VSD, PLSVC | ar11q24.2q25(126039017_134938470) ×1 | Deletion | 8.90 | P | TOP |
| 21 | 33.6 | 25.4 | VSD, PA | arr22q11.21(18648866_21465662) ×1 | Deletion | 2.82 | P | TOP |
| 22 | 31.5 | 23.6 | Isolated VSD | arr1q21.1q21.2(146488131_147819294) ×3 | Duplication | 1.33 | P | TOP |
| 23 | 27.8 | 31.3 | VSD, Choroid | arr16p13.11(15140210_16326223) ×3 | Duplication | 1.19 | P | TOP |
| 24 | 31.2 | 28.0 | VSD, HEK, | arr22q11.21(18648855-21800471) ×1 | Deletion | 3.15 | P | TOP |
| 25 | 33.2 | 24.7 | VSD, HEK, MCDK, FGR | arr10q23.33q26.3(96,180,463-135,427,143) ×3 arr12p13.33p13.32(173,786-4,843,935) ×1 | Duplication Deletion | 39.25  4.67 | P  P | TOP |
| 26 | 31.4 | 23.1 | VSD, corpus callosum agenesis | arr4p16.3p16.1(68345-721580) ×1 | Deletion | 8.65 | P | TOP |
| 27 | 25.1 | 21.3 | VSD, FGR | arr12q21.1q21.33(74290494-89992210) ×1 | Deletion | 15.70 | P | TOP |
| 28 | 32.1 | 23.5 | VSD, hydrothorax | Arr5p14.2p13.2(23869246 - 36948466) ×1 | Deletion | 13.08 | P | TOP |

HEK: hyperechogenic kidneys; CMA: chromosomal microarray analysis; MA: maternal age; GA: gestational age; CNV: copy number variant; P: pathogenic; TOP: termination of pregnancy; VSD: ventricular septal defect; AVSD: atrioventricular septal defect; PA: pulmonary atresia; MCDK: multiple cystic dysplastic kidneys; FGR: fetal growth restriction; PLSVC: persistent left superior vena cava.
